# Combined effects of insulin resistance and altitude of residence on 10-year risk of atherosclerotic cardiovascular disease

**DOI:** 10.3389/fcvm.2025.1656537

**Published:** 2025-11-26

**Authors:** Yu Xia, Liping He, Linhong Pang, Wenlong Zhu, Nan Zhang, Yue Gao, Lin Duo, Zhongjie Wang, Wenhui Yang, Mingjing Tang, Zhiling Luo, Da Zhu, Heng Su

**Affiliations:** 1 Fuwai Yunnan Hospital, Chinese Academy of Medical Sciences, Affiliated Cardiovascular Hospital of Kunming Medical University, Kunming, China; 2 School of Public Health, Kunming Medical University, Kunming, China; 3 School of Health Policy and Management, Chinese Academy of Medical Sciences and Peking Union Medical College, Beijing, China

**Keywords:** triglyceride–glucose index, altitude, atherosclerotic cardiovascular diseases, combined effect, risk

## Abstract

**Background:**

The combined health effects of the triglyceride–glucose (TyG) index and altitude of residence on the 10-year risk of atherosclerotic cardiovascular disease (ASCVD) remain unknown. We examined the combined effect of the TyG index and altitude of residence on ASCVD using data from two cross-sectional studies.

**Methods:**

We included 6,861 participants with no history of ASCVD from two cross-sectional studies and assessed their 10-year risk of ASCVD using the China-PAR model. We stratified the TyG index and altitude of residence and used multivariate-adjusted logistic models to estimate odds ratios (ORs) and 95% confidence intervals (CIs) for a high 10-year risk of ASCVD. Both additive and multiplicative effects were considered.

**Results:**

A total of 6,861 participants free of ASCVD at baseline were included in the analysis. Among them, 559 study participants were classified as being at high risk for ASCVD. Compared with participants with the lowest TyG index levels, those with the highest TyG index levels were more likely to be at high risk for ASCVD [(OR): 2.17, 95% C1: 1.67, 2.82]. Non-linear relationships were observed in the restricted cubic spline analyses. Altitude of residence was also associated with an increased risk of ASCVD [(OR): per standard deviation: 1.46, 95% Cl: 1.29, 1.65]. However, there was limited evidence of interaction between the TyG index and altitude of residence. Similar findings were observed in a series of sensitivity analyses.

**Conclusion:**

Both the TyG index and altitude of residence were positively associated with a high risk of ASCVD separately; however, there was no significant interaction between the associations.

## 1 Introduction

Cardiovascular diseases (CVDs) are associated with high morbidity and mortality rates and ran first among chronic non-communicable diseases in terms of fatalities (1). In China, atherosclerotic cardiovascular disease (ASCVD) constitutes the majority of CVD cases, accounting for 61% of cardiovascular deaths in 2016, an increase of 21% compared with 1990 (2), representing a substantial burden on the healthcare system. Therefore, early identification of people at risk of ASCVD—through effective screening measures, as well as the development of preventive and treatment strategies—is essential.

It is well known that insulin resistance (IR) is one of the most important biomarkers of diabetes mellitus (DM) (3), while DM itself represents one of the most significant health outcomes associated with insulin resistance. The global burden of diabetes continues to increase, with the prevalence rate in China increasing by 2.5 times over the last 30 years (4, 5). Current research also indicates that IR shares an equally strong association with CVD, a conclusion that has been validated through large-scale cohort studies and Mendelian randomization research (6, 7). Epidemiological and pathophysiological studies suggest that IR may contribute to the development and progression of CVD by affecting vascular endothelial function (8). Commonly used clinical methods for assessing IR include the hyperinsulinemic–euglycemic clamp technique and the homeostasis model assessment of insulin resistance, both of which are cumbersome and costly testing procedures (9). The triglyceride–glucose (TyG) index, calculated using fasting blood glucose (FBG) and triglyceride (TG) levels, has been reported to significantly correlate with IR and serves as a simple and reliable surrogate marker of IR. In addition, the correlation of the TyG index with ASCVD risk has been validated across various cohorts (10, 11).

High-altitude environments can have a significant impact on the cardiovascular system; however, the results remain conflicting. Some studies have reported that prolonged exposure to high altitude leads to a sustained increase in blood pressure, resulting in a higher risk of hypertension and cardiovascular disease (12, 13). However, other studies have found that blood pressure remained stable or even decreased (14, 15) in high-altitude environments, significantly reducing the risk of cardiovascular morbidity or mortality in these populations.

Previous studies have shown that long-term exposure to high-altitude hypoxia may impair vascular function (16), while the TyG index has also been associated with vascular damage and cardiovascular disease. However, it remains unclear how cardiovascular disease risk changes when both factors coexist in the same individual. Current research has not yielded definitive conclusions. Therefore, we propose the key hypothesis of this study: insulin resistance and altitude may interact to influence ASCVD risk, with the combined effect likely being positive. This study employed the reliable China-PAR model to assess the 10-year incidence risk of ASCVD among individuals without prior cardiovascular disease at baseline, exploring the independent and combined health effects of the TyG index and altitude of residence on ASCVD risk. These findings will aid in the early identification of high-risk populations and inform targeted interventions to reduce the burden of ASCVD.

## 2 Materials and methods

### 2.1 Study population and design

**Figure 1:**
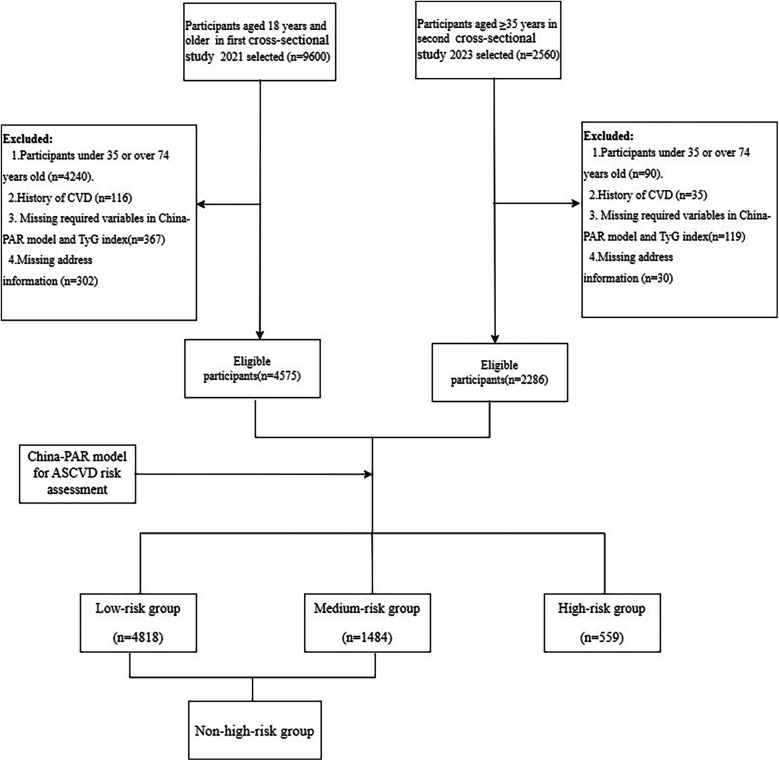
Flowchart of participants inclusion.

Both cross-sectional studies were conducted in accordance with local and national regulations and the principles of the Declaration of Helsinki. The first dataset was obtained from the “Chinese Resident Cardiovascular Disease and Risk Factor Surveillance Program,” carried out in Yunnan Province between January and December 2021. The sampling procedures and methods employed have been published previously (17). In summary, eight districts/counties were selected using a stratified multistage random sampling method to screen permanent residents aged 18 years for CVD. The second dataset was obtained from a survey on CVD-related risk factors among highland residents of Yunnan Province, conducted between September 2023 and January 2024. The specific sampling procedure is outlined in Supplementary Material Figure S1. In brief, stratified multistage random sampling was employed to select permanent residents aged 35 years and older from four townships located at altitudes exceeding 2,500 m in Yunnan Province for CVD screening. Both projects were approved by the Ethics Committee of Fuwai Hospital, Chinese Academy of Medical Sciences, and Fuwai Yunnan Cardiovascular Disease Hospital. All respondents provided informed consent. The two studies were integrated into a single database, with further exclusion of participants with a prior history of CVD and those with missing variables for ASCVD risk assessment, resulting in the inclusion of a total of 6,861 study participants aged 35–74 years. The participant inclusion process is presented in Figure 1.

### 2.2 Data collection and definitions

Both cross-sectional studies were conducted in township or community health centers using the same survey protocol and data collection forms. A survey team of 20 uniformly trained internists, sonographers, nurses, pharmacists, and medical students administered questionnaires, performed physical examinations, and collected blood samples from participants. The questionnaires collected information on age, gender, education level, per capita annual income, smoking status, alcohol intake status, physical activity, self-reported medical history (hypertension and diabetes mellitus), and medication use (glucose-lowering, antihypertensive, and lipid-lowering medications).

Education levels were divided into two groups: primary school and below and junior high school and above. Low income was defined as an annual income of ≤ 20,000 RMB. Current smokers were defined as individuals who smoked at least one cigarette a day for more than 6 months, and current drinkers were defined as those who consumed alcohol at least once a week in the last year. According to the World Health Organization (WHO) 2020 Guidelines on Physical Activity and Sedentary Behaviour (18), physical inactivity was defined as engaging in ≤150 min of moderate-intensity physical activity or <75 min of vigorous-intensity physical activity per week. Insufficient intake of vegetables and fruits was defined as consuming them on fewer than 3 days/month (19). Overweight was defined as BMI = 24.0–27.9 kg/m^2^ and obesity as BMI ≥ 28 kg/m^2^ (20). Hyperuricemia was defined as serum uric acid levels of >420 μmol/L in men and >360 μmol/L in women (21).

Systolic blood pressure (SBP) and diastolic blood pressure (DBP) were measured using an OMRON HBP-1300 electronic sphygmomanometer. Hypertension was defined as SBP ≥ 140 mmHg and/or DBP ≥ 90 mmHg, self-reported hypertension, or self-reported use of antihypertensive medications within the last 2 weeks (22). Fasting blood samples were collected by vein puncture in the morning after at least 8 h of fasting. Total cholesterol (TC), triglyceride (TG), high-density lipoprotein cholesterol (HDL-C), low-density lipoprotein (LDL), fasting blood glucose, serum creatinine, and blood uric acid levels were measured. The eGFR was calculated using a modified Chinese version of the Modification of Diet in Renal Disease equation: eGFR = 175 × Scr^−1.234^ × age^−0.179^ [×0.79 if female], where Scr denotes serum creatinine concentration (mg/dL) and age denotes the age of the participant. Chronic kidney disease was defined as eGFR <  mL/min/1.73 m^2^) (23). Diabetes mellitus was defined as fasting blood glucose ≥7.0 mmol/L or a self-reported history of diabetes mellitus. Hypercholesterolemia (24) was defined as a self-reported history of dyslipidemia or TC > 5.17 mmol/L.

The TyG index was calculated as lnTG [(mg/dL) × FBG (mg/dL)/2] (11, 24). The China-PAR model was employed as recommended by “the Chinese Guidelines for Cardiovascular Disease Risk Assessment and Management.” The model has been demonstrated to exhibit satisfactory internal consistency and external validity (25). The China-PAR model, incorporating suitable variables, was used to calculate the 10-year ASCVD risk stratification. A risk classification of <5% was considered a low risk, 5%–9% a moderate risk, and ≥10% a high risk. Detailed information of the 11 variables included in the model is provided in the Supplementary Material.

### 2.3 Statistical analyses

Continuous variables were expressed as means and standard deviations (SDs) for normally distributed data and as medians with interquartile ranges for non-normally distributed data. Categorical variables were presented as frequencies and percentages. The chi-square test, ANOVA, or Kruskal–Wallis rank-sum test was utilized to compare characteristics between groups. Multiple logistic regression models were employed to examine the association between different levels of the TyG index, altitude of residence, and high ASCVD risk. By adding covariates (demographic characteristics, socioeconomic factors, and lifestyle factors), we built a four-model analytical framework. Models 1, 2, and 3 were first constructed to test the independent association of the TyG index with altitude. Model 4 was then constructed for mutual adjustment (protocol details are given in the Supplementary Material). Odds ratios (ORs) and 95% confidence intervals (95%CIs) were calculated for each interquantile range (IQR) increment in the TyG index (0.89) and per 1-SD (631.4 m) in altitude. Categorical exposures were calculated with reference to the lowest level of the TyG index or the first tertile of altitude. Potential non-linear associations were calculated using four-knot restricted cubic spline regression. Subgroup analyses were performed using Model 3, stratified by the TyG index (<8.12, 8.12–8.54, 8.54–9.01, and >9.01) or altitude (<2,000 m, 2,000–2,999 m, and ≥3,000 m). To further explore the existence of a linear trend between the TyG index or altitude and the high risk of ASCVD, we assigned a median value to each group.

The study further investigated the potential interactions between the TyG index and altitude exposure on both additive and multiplicative scales. For additive interaction, we first categorized study participants into four groups based on their TyG index. Combined with altitude levels (three groups), we then created a new term with 12 categories representing 12 combinations (4 × 3) of TyG index exposure levels and altitude levels for testing additive interaction (26). Next, we calculated the relative excess risk due to interaction (RERI) and corresponding confidence intervals (Cls) using participants in the lowest level of TyG index quartile (Q1) and altitude <2,000 m as the reference group. RERI measures the combined excess risk in both exposed groups that arises from their interaction. A RERI score of 0 denotes no additive interaction (the combined excess risk is the sum of their individual excess risks, calculated as OR-1). A RERI greater than 0 indicates a positive interaction (meaning that the combined excess risk is more than the sum of their individual excess risks), while a RERI less than 0 implies a negative interaction (meaning that the combined excess risk was less than the sum of their individual excess risks. Therefore, a positive RERI value in the present study indicates that residential altitude amplifies the ASCVD risk associated with the TyG index. On the multiplicative scale (27), we added a product term between the TyG index and altitude levels. Likelihood tests were employed to assess the significance of this interaction by comparing the model with and without the interaction term. A *p*-value of less than 0.05 for the interaction term was considered indicative of a multiplicative interaction.

### 2.4 Sensitivity analyses

(1) We excluded participants who were treated with glucose-lowering medications. (2) We also excluded participants treated with lipid-lowering medications. (3) We calculated *E*-values to evaluate the potential impact of unmeasured confounders on conclusions in observational studies (28, 29). Details can be found at https://www.evalue-calculator.com/.

All analyses were performed using R version 4.3.0. A two-sided *p* value of less than 0.05 was considered statistically significant.

## 3 Results

### 3.1 Characteristics of participants

A total of 6,861 participants were included in the study. The mean age of participants was 52.2 ± 10.5 years and 3,191 (46.5%) were men. After assessment using the China-PAR model, the mean 10-year ASCVD risk for the 6,861 study participants was 2.7 (1.1,5.7), of which 559 (8.1%) were classified as high risk, 1,484 (21.6%) as medium risk. The characteristics of participants stratified by ASCVD risk scores are presented in Table 1. Participants in the high-risk group were predominantly Han Chinese, male, current smokers, and exhibited higher rates of alcohol consumption and physical inactivity. They also tended to have lower education and lower income levels. Additionally, this group showed a higher prevalence of hypertension, diabetes mellitus, chronic kidney disease, and hypercholesterolemia, as along with higher levels of SBP, DBP, FBG, and TC. The distribution of ASCVD risk across participants with TyG and altitude level changes is presented in Supplementary Material Figure S2 and Supplementary Table S1.

**Table 1:** Basic characteristics of participants with stratified ASCVD risk.

Variables	Overall (*n* = 6,861)	Non-high-risk group		High-risk group (*n* = 559)	*p-*Value
		Low-risk (*n* = 4,818)	Medium-risk (*n* = 1,484)		
Age, years	52.2 ± 10.5	48.1 ± 8.3	60.9 ± 8.4	63.8 ± 8.7	<0.001
Sex					<0.001
Male	3,191 (46.5)	2,012 (41.8)	789 (53.2)	390 (69.8)	
Female	3,670 (53.5)	2,806 (58.2)	695 (46.8)	169 (30.2)	
Ethnic					<0.001
Han	3,452 (50.3)	2,512 (52.3)	654 (44.1)	286 (51.2)	
Minority	3,409 (49.7)	2,306(47.7)	830 (55.9)	273 (48.8)	
Education					<0.001
Primary school and below	3,692 (53.8)	2,339 (48.5)	975 (65.7)	378 (67.6)	
Junior high school and above	3,169 (46.2)	2,479 (51.5)	509 (34.3)	181 (32.4)	
Income					<0.001
≤20,000 RMB/year	4,024 (58.7)	2,718 (56.4)	943 (63.5)	363 (64.9)	
>20,000 RMB/year	2,837 (41.3)	2,100 (43.6)	541 (36.5)	196 (35.1)	
Residence					0.001
Urban	3,962 (57.7)	2,813 (58.4)	878 (59.2)	271 (48.5)	
Rural	2,899 (42.3)	2,005 (41.6)	606 (40.8)	288 (51.5)	
Current smoking	1,815 (26.5)	1,138 (23.6)	459 (30.6)	218 (39.0)	<0.001
Current drinking	833 (12.1)	470 (9.8)	235 (15.8)	128 (22.9)	<0.001
Insufficient physical activity	3,102 (45.2)	2,117 (43.9)	702 (47.3)	283 (50.6)	0.002
Insufficient vegetables and fruits	3,476 (50.7)	2,429 (50.4)	742 (50.0)	305 (54.6)	0.197
Hypertension	2,891 (42.1)	1,318 (27.4)	1,086 (73.2)	487 (87.1)	<0.001
DM	581 (8.5)	213 (4.4)	190 (12.8)	178 (31.8)	<0.001
Chronic kidney disease	288 (4.2)	136 (2.8)	106 (7.1)	46 (8.2)	<0.001
Hyperuricemia	1,488 (21.7)	933 (19.4)	377 (25.4)	178 (31.8)	<0.001
Hypercholesterolemia	2,786 (40.6)	1,775 (36.8)	721 (48.6)	290 (51.9)	<0.001
Glucose-lowering medication	289 (4.2)	97 (2.0)	97 (6.5)	95 (17.0)	<0.001
Hypertension medications	1,202 (17.5)	358 (7.4)	554 (37.3)	290 (51.9)	<0.001
Lipid-lowering medication	132 (1.9)	69 (1.4)	45 (3.0)	18 (3.2)	<0.001
BMI, kg/m^2^	24.1 ± 3.6	23.9 ± 3.5	24.5 ± 3.9	24.9 ± 3.9	<0.001
SBP, mmHg	131.5 ± 19.6	125.0 ± 15.8	143.1 ± 17.5	156.2 ± 20.6	<0.001
DBP, mmHg	81.7 ± 11.6	79.5 ± 10.4	85.9 ± 12.0	90.5 ± 13.1	<0.001
FBG, mg/dL	95.5 ± 30.1	92.1 ± 30.1	100.1 ± 30.2	113.4 ± 30.2	<0.001
TG^a^, mg/dL	111.6 (76.1,169.2)	106.7 (73.5,163.9)	120.5 (83.3,177.2)	129.4 (84.2,210.9)	<0.001
TC, mg/dL	193.1 ± 42.1	190.1 ± 40.5	199.4 ± 44.4	202.0 ± 46.1	<0.001
HDL-C, mg/dL	57.3 ± 14.9	57.8 ± 14.9	56.8 ± 15.1	54.1 ± 14.1	<0.001
LDL-C, mg/dL	112.1 ± 33.0	109.9 ± 32.2	116.4 ± 34.0	119.5 ± 35.0	<0.001

ASCVD, atherosclerotic cardiovascular diseases; DM, diabetes mellitus; BMI, body mass index; SBP, systolic blood pressure; DBP, diastolic blood pressure; FBG, fasting blood glucose; TC, total cholesterol; TG, triglyceride; HDL-C, high-density lipoprotein cholesterol; LDL-C, low-density lipoprotein cholesterol.

a Medians and quartiles are reported for non-normally distributed continuous variables.

### 3.2 Association between the TyG index and a high risk of ASCVD

**Figure 2:**
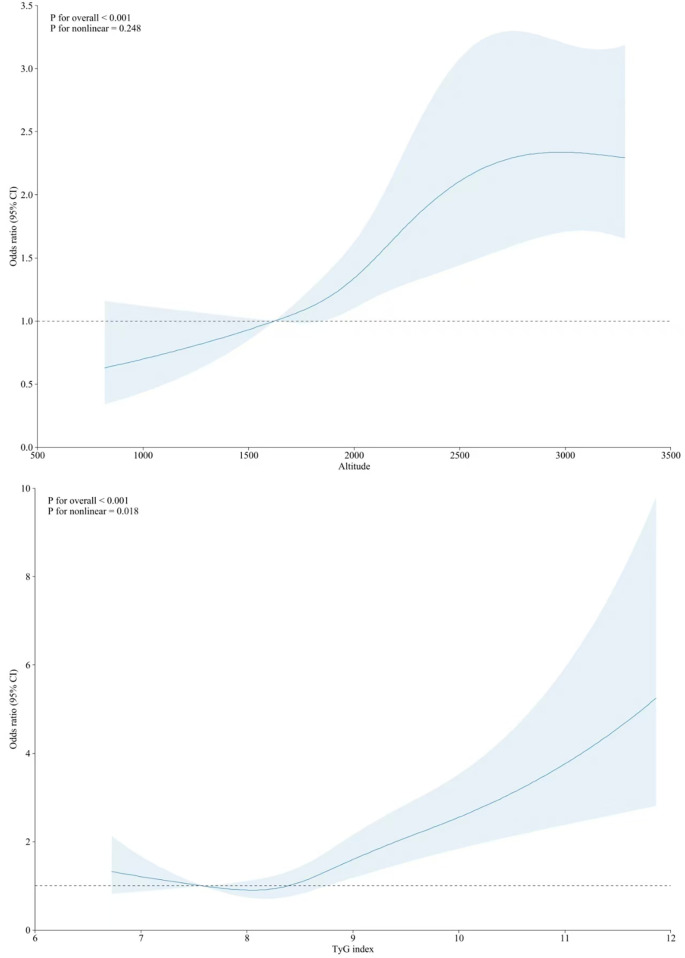
Nonlinear associations of altitude (top) and TyG index with10-year high risk of ASCVD (bottom).

Table 2 shows the associations between the TyG index level and a high risk of ASCVD. A higher TyG index level was associated with an increased risk of high ASCVD. These associations remained robust after adjustment for various covariates, including altitude level. In Model 4, compared with the Q1 TyG index level, the ORs (95% CIs) for high ASCVD risk were 1.04 (95% CI: 0.79,1.38) for Q2, 1.43 (95% CI: 1.10,1.87) for Q3, and 2.19 (95% CI: 1.67,2.82) for Q4. The non-linear associations between the TyG index and high risk of ASCVD are shown in Figure 2. In subgroup analyses, the positive association between the TyG index level and high risk of ASCVD remained stable across participants residing at different altitude levels (Table 3).

**Table 2:** Association of the TyG and increasing altitude with the high risk of ASCVD.

Levels		Model 1^b^ OR (95% CI)	Model 2^b^ OR (95% CI)	Model 3^b^ OR (95% CI)	Model 4^b^ OR (95% CI)
TyG^a^	Events				
Q1	91	Reference	Reference	Reference	Reference
Q2	99	1.12 (0.85–1.48)	1.09 (0.83–1.44)	0.99 (0.75–1.31)	1.04 (0.79–1.38)
Q3	144	**1.67** (**1.29–2.15)**	**1.57** (**1.22–2.02)**	**1.32** (**1.01–1.71)**	**1.43** (**1.10–1.87)**
Q4	225	**2.83** (**2.23–3.58)**	**2.59** (**2.04–3.29)**	**1.95** (**1.51–2.26)**	**2.17** (**1.67–2.82)**
*P* for trend		<0.001	<0.001	<0.001	<0.001
Per IQR (0.89) increment		**1.60** (**1.47–1.73)**	**1.58** (**1.46–1.71)**	**1.33** (**1.21–1.46)**	**1.36** (**1.23–1.49)**
Altitude					
<2,000 m	250	Reference	Reference	Reference	Reference
2,000–2,999 m	142	**1.47** (**1.20–1.79)**	**1.46** (**1.22–1.81)**	**1.51** (**1.23–1.85)**	**1.51** (**1.23–1.85)**
≥3,000 m	167	**2.20** (**1.69–2.86)**	**2.32** (**1.63–2.80)**	**2.32** (**1.76–3.15)**	**2.59** (**1.97–3.41)**
*P* for trend		<0.001	<0.001	<0.001	<0.001
Per1-SD (631.4 m) increment		**1.37** (**1.22–1.54)**	**1.42** (**1.26–1.60)**	**1.41** (**1.25–1.60)**	**1.46** (**1.29–1.65)**

The bold type represents the statistically significant differences (*p* < 0.05). The dependent variable in all models is a binary variable, high-risk group vs. non-high-risk group, with the non-high-risk group serving as the reference group.

a TyG, triglyceride–glucose; OR, odds ratio; CI, confidence interval; ASCVD, atherosclerotic cardiovascular disease; Q1, [5.47–8.12]; Q2, (8.12–8.54]; Q3, (8.54–9.01]; Q4, (9.01–13.17].

b Model 1 was adjusted for education, ethnicity, and income. Model 2 was further adjusted for alcohol drinking, insufficient physical activity, and insufficient consumption of vegetables and fruits. Model 3 was further adjusted for overweight or obesity, LDL-C, hyperuricemia, chronic kidney disease, lipid-lowering medications, and glucose-lowering medications. Model 4 was further mutually adjusted for the TyG index (effect estimation of altitude) or altitude (effect estimation of TyG).

**Table 3:** Association between TyG index levels and high risk of ASCVD when individuals are exposed to different altitude levels.

TyG index levels^a^	Events	<2,000 m Level	*P*	2,000–2,999 m Level	*P*	≥3,000 m Level	*P*
		OR (95% CI)^b^		OR (95% CI)^b^		OR (95% CI)^b^	
Q1	91	Reference		Reference		Reference	
Q2	99	1.05 (0.63–1.76)	0.858	1.17 (0.63–2.15)	0.621	1.07 (0.71–1.61)	0.749
Q3	144	1.51 (0.95–2.40)	0.081	1.28 (0.72–3.00)	0.400	1.53 (0.99–2.35)	0.056
Q4	225	**2.48** (**1.59–3.86)**	<0.001	**2.73** (**1.56–4.78)**	<0.001	**1.62** (**1.01–2.62)**	0.048
*P* for trend			<0.001		<0.001		0.011

The bold type represents the statistically significant differences (*p* < 0.05).

The dependent variable in all models is a binary variable, high-risk group vs. non-high-risk group, with the non-high-risk group serving as the reference group.

a TyG, triglyceride–glucose; OR, odds ratio; CI, confidence interval; ASCVD, atherosclerotic cardiovascular disease; Q1, [5.47–8.12]; Q2, (8.12–8.54]; Q3, (8.54–9.01]; Q4, (9.01–13.17].

b Model 1 was adjusted for education, ethnicity, and income. Model 2 was further adjusted for alcohol drinking, insufficient physical activity, and insufficient consumption of vegetables and fruits. Model 3 was further adjusted for overweight or obesity, LDL-C, hyperuricemia, chronic kidney disease, lipid-lowering medications, and glucose-lowering medications.

### 3.3 Association between the altitude level and a high risk of ASCVD

Altitude was also positively associated with a higher risk of ASCVD, and this correlation remained unchanged after adjusting for the TyG index and other covariates (Table 2). Every 1-SD increase in altitude was associated with a 46.0% higher risk of ASCVD. In the tertile-based analysis, compared with participants residing at altitudes <2000 m, the ORs for a high risk of ASCVD were 1.51 (95% CI: 1.23,1.85) and 2.86 (95% CI: 1.97,3.41) for those living at 2,000–2,900 and ≥3,000 m (Table 2), respectively. The non-linear associations between altitude and a high risk of ASCVD are presented in Figure 2. The positive association persisted across subgroups with different TyG index levels, although some results were statistically non-significant (Table 4).

**Table 4:** Association between altitude levels and high risk of ASCVD when individuals were exposed to different TyG index levels.

Altitude levels	Events	Q1-level TyG^a^	*P*	Q2-level TyG^a^	*P*	Q3-level TyG^a^	*P*	Q4-level TyG^a^	*P*
		OR (95% CI)^b^		OR (95% CI)^b^		OR (95% CI)^b^		OR (95% CI)^b^	
<2,000 m	250	Reference		Reference		Reference		Reference	
2,000–2,999 m	142	1.77 (0.94–2.96)	0.076	1.60 (0.93–2.75)	0.088	1.37 (0.90–2.09)	0.145	**1.58** (**1.18–2.11)**	0.002
≥3,000 m	167	**3.19** (**1.48–6.87)**	0.003	**3.12** (**1.64–5.94)**	0.001	**3.00** (**1.76–5.13)**	<0.001	**2.19** (**1.35–3.36)**	0.002
*P* for trend			<0.001		<0.001		<0.001		<0.001

The bold type represents the statistically significant differences (*p* < 0.05).

The dependent variable in all models is a binary variable, high-risk group vs. non-high-risk group, with the non-high-risk group serving as the reference group.

a TyG, triglyceride–glucose; OR, odds ratio; CI, confidence interval; ASCVD, atherosclerotic cardiovascular disease; Q1, [5.47–8.12]; Q2, (8.12–8.54]; Q3, (8.54–9.01]; Q4, (9.01–13.17].

b Model 1 was adjusted for education, ethnicity, and income. Model 2 was further adjusted for alcohol drinking, insufficient physical activity, and insufficient consumption of vegetables and fruits. Model 3 was further adjusted for overweight or obesity, LDL-C, hyperuricemia, chronic kidney disease, lipid-lowering medications, and glucose-lowering medications.

### 3.4 Potential interaction between TyG index levels and altitude levels on the high risk of ASCVD

Table 5 presents the combined health impact of TyG index and altitude levels on ASCVD risk. Participants with higher TyG index levels and high altitude exposure generally exhibited a higher risk of ASCVD. Using participants with low TyG index levels and low altitude exposure as the reference group, those with high TyG index levels and high altitude exposure demonstrated the highest risk of ASCVD (OR: 4.95,95% CI: 2.84,8.63). The results of the interaction between TyG index levels and altitude levels on the high risk of ASCVD are presented in Table 5. On the additive scale, little evidence of interaction was found. Similar results were also observed on the multiplicative scale, with *p*-values >0.05 for the interaction term (Supplementary Material Table S2).

**Table 5:** Combined effects of TyG index levels and altitude levels on high risk of ASCVD on additive scales.

TyG index levels^a^	Altitude levels (OR, 95% CI)^b^			RERI	
	<2,000 m level	2,000–2,999 m level	≥3,000 m level	2,000–2,999 m level	≥3,000 m level
Q1	Reference	1.67 (0.91–3.09)	**3.20** (**1.94–5.28)**		
Q2	1.09 (0.650–1.82)	**1.90** (**1.10–3.23)**	**3.28** (**1.93–5.59)**	0.14 (−0.40,0.67)	0.02 (−0.32,0.36)
Q3	**1.67** (**1.06–2.64)**	**2.08** (**1.24–3.48)**	**4.69** (**2.81–7.85)**	0.17 (−0.32,0.66)	0.31 (−0.11,0.72)
Q4	**2.65** (**1.72–4.09)**	**4.12** (**2.61–6.53)**	**4.95** (**2.84–8.63)**	0.31 (−0.12,0.75)	0.33 (−0.07,0.73)

The bold type represents the statistically significant differences (*p* < 0.05).

The dependent variable in all models is a binary variable, high-risk group vs. non-high-risk group, with the non-high-risk group serving as the reference group.

a TyG, triglyceride–glucose; OR, odds ratio; CI, confidence interval; ASCVD, atherosclerotic cardiovascular disease; Q1, [5.47–8.12]; Q2, (8.12–8.54]; Q3, (8.54–9.01]; Q4, (9.01–13.17].

b Model 1 was adjusted for education, ethnicity, and income. Model 2 was further adjusted for alcohol drinking, insufficient physical activity, and insufficient consumption of vegetables and fruits. Model 3 was further adjusted for overweight or obesity, LDL-C, hyperuricemia, chronic kidney disease, lipid-lowering medications, and glucose-lowering medications.

### 3.5 Subgroup and sensitivity analyses

Subgroup analysis indicated that the association between the TyG index, altitude, and ASCVD risk remained consistent, but the effects were differential across different subpopulations and no significant interactions were observed (Supplementary Material Tables S3, S4). In sensitivity analyses, the associations of TyG index levels and altitude levels with high risk of ASCVD were not materially altered after excluding participants who received treatment with lipid-lowering medications or glucose-lowering medications (Supplementary Material Tables S5, S6). The *E*-values demonstrated that the associations of TyG index levels and altitude levels with a high risk of ASCVD were robust (Supplementary Material Table S7).

## 4 Discussion

To our knowledge, this is the first study to analyze the combined health effects of the TyG index and altitude of residence on ASCVD risk. We found that the TyG index and residential altitude were both independently associated with a high risk of ASCVD. However, there was little evidence of the interaction between the TyG index and altitude of residence on either additive or multiplicative scales.

The TyG index has been proposed as a simple yet powerful alternative indicator of IR and metabolic health for clinical applications (3). Studies have demonstrated that the TyG index was strongly associated with ASCVD, including coronary artery disease (30) and peripheral arterial disease (31). Moreover, several studies have revealed the association between the TyG index and risks of hypertension, myocardial infarction, and ischemic stroke (IS) (32). The TyG index and its derived parameters improved the predictive performance in the classical Framingham cardiovascular risk model (33). Other reports have further confirmed its clinical value in predicting ASCVD risk (34, 35). High levels of the TyG index may reflect underlying metabolic dysfunction, such as altered lipid exchange and lipolysis, leading to increased inflammatory response, endothelial dysfunction, and plaque instability, ultimately causing an increased risk of ASCVD (25). In our study, those with the highest levels of the TyG index demonstrated a 2.67 times higher risk of ASCVD than those with the lowest levels. Our results are similar to those of a more recent 9-year cohort study, which indicated that long-term high exposure to TyG index levels significantly increased the risk of stroke (aHR: 1.30,95% CI: 1.12,1.52) (32).

The association between altitude of residence and ASCVD risk has been a topic of controversy. A large Swiss cohort study suggested that high altitude was beneficial in reducing the risk of death from coronary heart disease and stroke (36). Studies on populations in the Andes and the Peruvian Plateau have also illustrated the beneficial effects of high altitude on the cardiovascular system (37, 38). Hypoxia reportedly had a direct diastolic impact on vascular smooth muscle cells, leading to vasodilation and a decrease in vascular resistance (39). However, our study found that the high risk of ASCVD appeared to increase linearly with rising altitude of residence, with a 45.9% increase in high risk of ASCVD per 1-SD increase. Consistent with other studies (40, 41), previous findings have suggested that the hypoxic environment of high-altitude residence causes activation of the centrally mediated adrenergic system with vasoconstriction effects on peripheral *α*-adrenergic receptors, which can lead to an increase in peripheral vascular resistance and blood pressure (37, 39). Another important theory suggests that differences in cardiovascular adaptations because of racial disparity may be responsible for these contradictory findings (39). In addition, our study identified unique dietary patterns among high-altitude residents, characterized by a diet predominantly consisting of beef and mutton, with high fat, high calorie, and high salt intake, accompanied by low intake of vegetables and fruits and a less physically active lifestyle. These aspects contribute to a cluster of cardiovascular disease risk factors, such as obesity and elevated blood pressure, which increase the risk of ASCVD (42). The study also reported similar findings to previous research, indicating that high-altitude populations tend to exhibit higher HDL levels and lower total cholesterol or LDL levels (43). Nevertheless, altitude is a complex exposure factor, as the unique altitude environmental trends shape distinctive dietary and lifestyle patterns among plateau populations, leading to certain physiological adaptive changes. These alterations exert a series of complex effects on cardiovascular health in high-altitude populations (37, 44). However, the benefits and adverse impacts of these effects remain contradictory and warrant further investigation in the future.

This study investigated the association between combined exposure to the TyG index and altitude of residence with a high risk of ASCVD. We attempted to associated the TyG index with altitude level to present a more accurate prediction of ASCVD risk than the two individual factors. We found that their combined effect did not exceed the sum of their individual effects. The positive associations between altitude of residence and ASCVD risk remained stable regardless of the TyG index, with the reverse also holding true. Although the additive and multiplicative effects were not statistically significant, nearly all results showed positive trends. Furthermore, among the 12 combinations, the combination with the highest TyG index and greatest altitude exposure showed the strongest association with ASCVD risk, which may underscore the practical significance of both factors on ASCVD risk. With approximately 330 million people affected, the incidence of CVD is rising in China. In 2019, ASCVD accounted for 22% of total deaths and 245.5 million disability-adjusted life years (45). Therefore, the results of this study will aid in future ASCVD prevention, particularly in those with high TyG index levels living in high-altitude regions. Although it is not easy to change their altitude of residence, individuals can reduce their TyG index by adopting a healthy lifestyle, thereby reducing the risk of ASCVD.

In subgroup analyses, we found that the TyG index and altitude showed inconsistent effects on ASCVD risk. The impact of the TyG index was more pronounced in women and younger individuals. Previous studies have indicated that compared to men, women have an increased risk of impaired glucose tolerance and exhibit greater insulin sensitivity (46). Although the protective role of estrogen against type 2 diabetes and insulin resistance in women has been known, this effect is limited to the premenopausal period, as women lose this protection and become more susceptible to metabolic disorders in the postmenopausal period (47). However, a US population study indicated that the association between the TyG index and 10-year ASCVD risk is stronger in men than in women (48). In future research, it is essential to conduct more in-depth investigations into sex differences influencing insulin resistance and its clinical outcomes. In contrast, altitude appears to have a greater effect on men, older adults, ethnic minority groups, and individuals with chronic diseases such as hypertension and DM. This disparity may stem from the more severe natural survival challenges (extreme cold, hypoxia), less healthy lifestyle patterns (high salt, high fat intake), and limited access to medical resources among high-altitude populations. Collectively, these adverse conditions increase ASCVD risk.

In summary, our study offers several advantages. First, it innovatively explored the combined effects to the TyG index and altitude of residence on the high risk of ASCVD. Second, while the association between altitude and ASCVD risk has remained controversial, and is particularly unclear in China, the present study further explored the association of altitude with an increased risk of ASCVD. Third, our study identified people at high risk of ASCVD and adjusted for the many available confounders—including demographic, behavioral, and metabolic characteristics and drug use—by performing multiple sensitivity analyses.

However, our study has certain limitations that warrant consideration. First, although we adjusted for numerous potential confounders, the causal inference of the TyG index and altitude of residence on high ASCVD risk was limited by the cross-sectional study design. Therefore, the results should be viewed with caution. Moreover, our study population was restricted to Yunnan Province, China, which could affect the generalizability of the results. Second, HbAlc and the 2-h oral glucose tolerance test were not measured but rather estimated using fasting blood samples, which might have overestimated or underestimated the actual prevalence of diabetes mellitus and the risk of ASCVD. Third, psychosocial factors (such as stress, depression, and adverse childhood experiences) and behavioral factors (sleep quality) are closely related to cardiovascular disease. However, this study did not collect information on these relevant variables, which may affect the stability of the results. Finally, key variables in the China-PAR assessment model—such as the use of antihypertensive medication, history of diabetes mellitus, and family history of cardiovascular disease—were self-reported by the study participants, which may have introduced a potential recall bias.

## 5 Conclusions

In conclusion, based on two cross-sectional studies, we found that residential altitude was associated with a high risk of ASCVD. Regardless of the altitude level, the TyG index was also associated with a high risk of ASCVD, indicating that the TyG index and altitude independently influence the high risk of ASCVD.

## Data Availability

The raw data supporting the conclusions of this article will be made available by the authors, without undue reservation.
