# Supplementary material for: Combined effects of insulin resistance and altitude of residence on 10-year risk of atherosclerotic cardiovascular disease
Source: Front Cardiovasc Med. 2025 Nov 26;12:1656537. doi: 10.3389/fcvm.2025.1656537 (PMC12689511; doi:10.3389/fcvm.2025.1656537)
Supplement: Supplementary file 1 [file Datasheet1.pdf]

Supplementary Material

Combined effect of insulin resistance and altitude of residence on 10 years risk of atherosclerotic cardiovascular diseases

China-PAR model risk assessment

The China-PAR model incorporates the following variables: sex, age, current place of residence (urban/rural, southern/northern), waist circumference, TC, HDL-C, current diastolic and systolic blood pressure levels, whether or not antihypertensive medications are being taken, whether or not diabetes mellitus is present, whether or not current smoking is occurring, and whether or not a family history of cardiovascular disease is present.

4-model analytical protocol:

We developed a 4-model analysis protocol by adding covariates(demographic characteristics, socioeconomic factors, lifestyle factors) incrementally, We investigated the independent associations of TyG and altitude first by building Model 1, 2 and 3. Then Model 4 was constructed for mutual adjustment. Model 1 was adjusted for Education, Ethnicity, Income. Model 2 was further adjusted for alchol drinker, lnsuficient physical activity,lnsuficient vegetables and fruits, Model 3 was further adjusted for Overweight or obese, LDL-C, Hyperuricaemia, Chronic kidney disease, lipid-lowering medication, glucose-lowering medication. And Model 4 was further mutually adjusted by TyG (effect estimation of altitude;) or altitude (effect estimation of TyG)

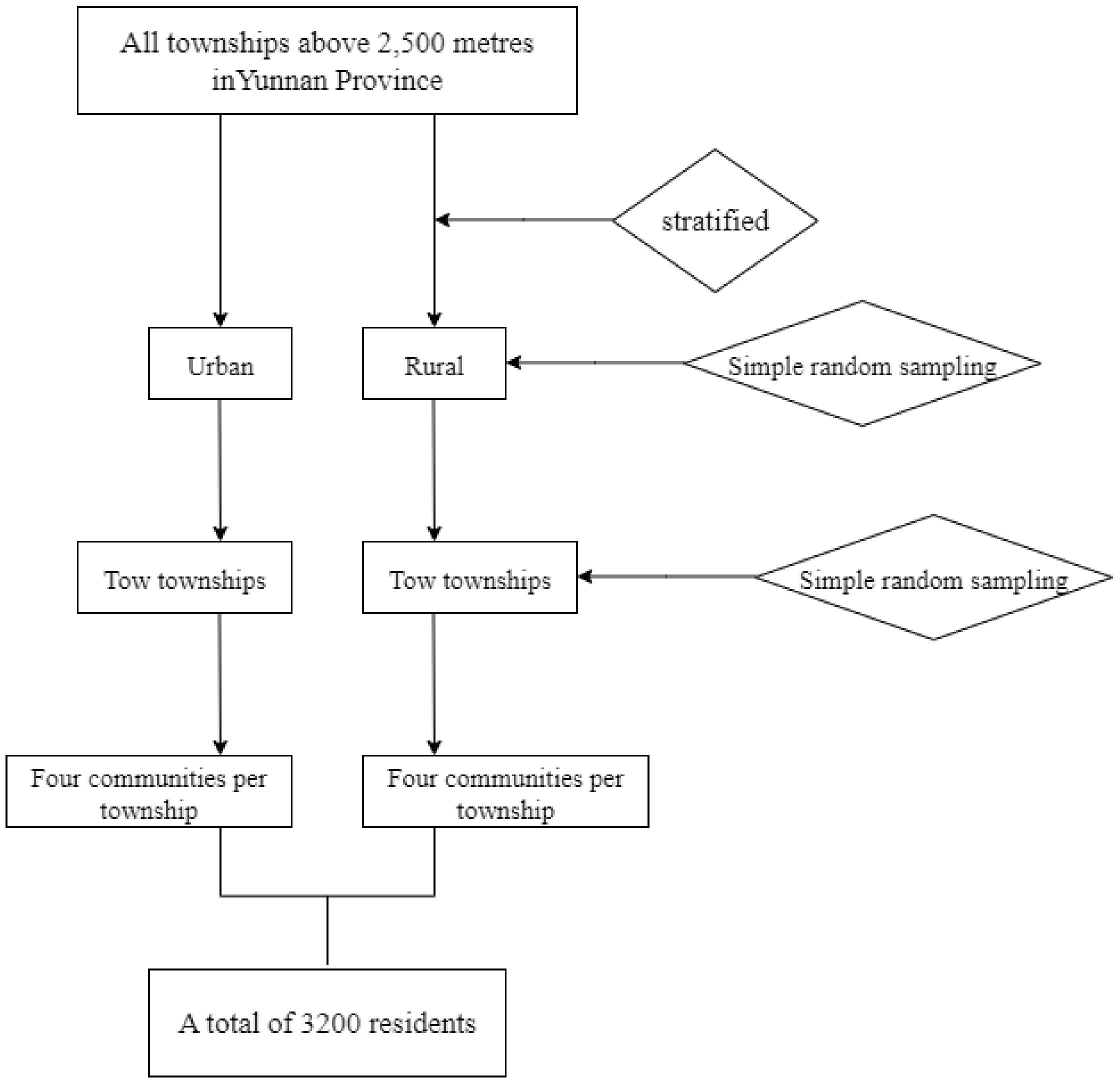

Figure S1 Sampling process for the second cross-sectional study

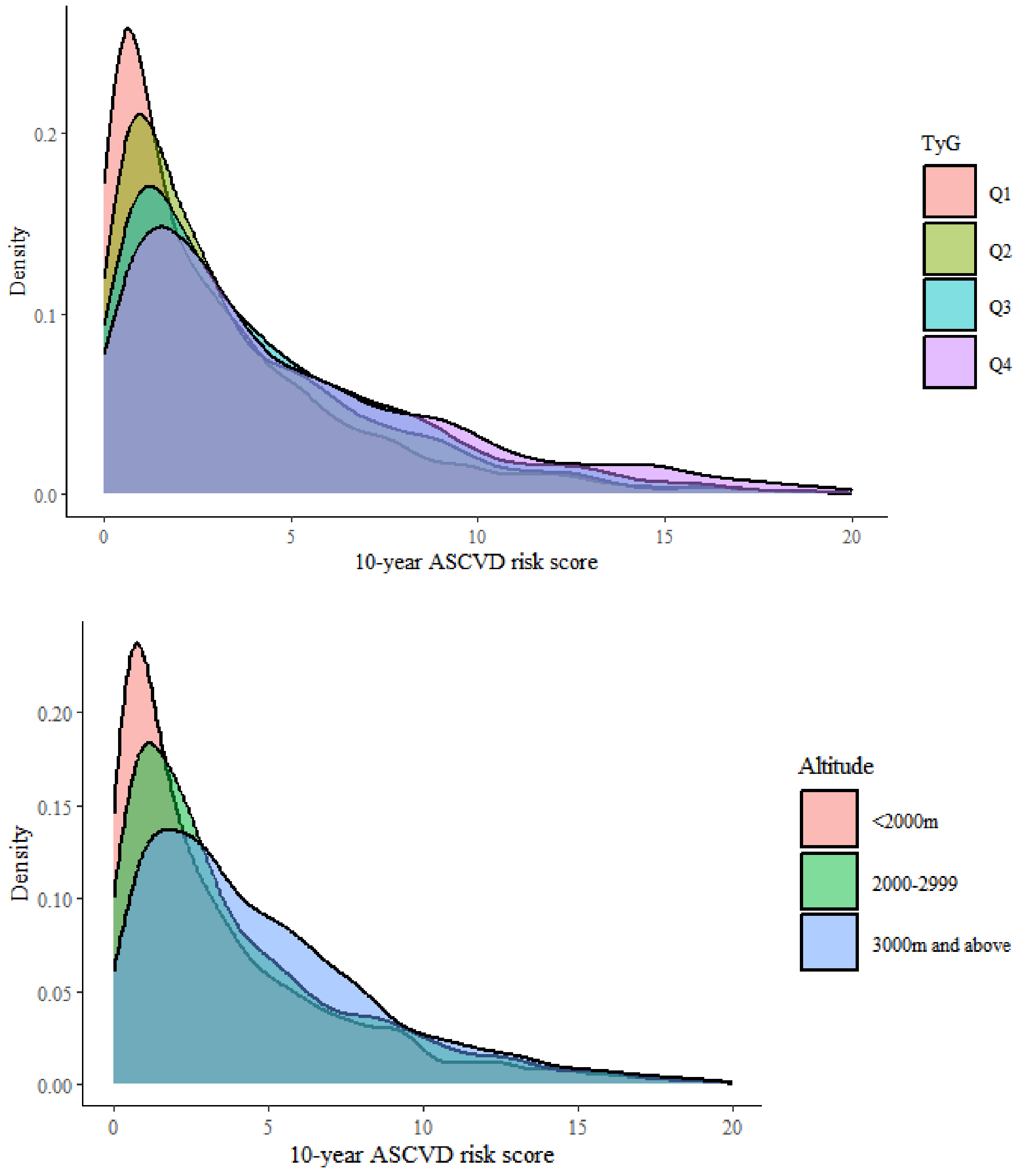

Figure S2 Distribution of ASCVD risk by subgroups of different TyG levels(Top) and altitude levels(Bottom)

Table S1 ASCVD risk scores for different TyG and altitude levels

Levels	ASCVD Risk scores [2.7(1.1, 5.7)]	Non-high-risk group		High-risk group (n=599)
		Low-risk (n=4818)	Medium-risk (n=1484)	
**TyG**[#a]				
Q1	2.0(0.7, 4.5)	1351(78.4)	282(16.4)	91(5.2)
Q2	2.4(1.0, 5.2)	1254(73.2)	360(21.0)	99(5.8)
Q3	3.1(1.3, 6.2)	1164(67.5)	417(24.2)	144(8.3)
Q4	3.4(1.6, 7.2)	1049(61.7)	425(25.0)	225(13.2)
**Altitude**[#]				
<2000	0.8(2.2, 5.1)	2763(74.3)	705(19.0)	250(6.7)
2000-2999	1.2(2.7, 5.7)	1135(70.0)	344(21.2)	142(8.8)
≥3000	1.9(3.8, 6.8)	920(60.4)	435(28.6)	167(11.0)

[#]Linear-by-Linear Association test, $P<0.05$

a:Abbreviations: TyG, triglyceride-glucose, Q1,[5.47-8.12], Q2, (8.12-8.54], Q3, (8.54-9.01], Q4, (9.01-13.17]

Table S2 Combined effects of TyG levels and altitude levels on high risk of ASCVD on multiplicative scales

TyG Levels[a]	Altitude Levels			*P*-interaction
	<2000m-Level	2000-2999m-Level	≥3000m-Level	
Q1	Reference			
Q2		1.07(0.46-2.47)	1.01(0.59-2.00)	0.521
Q3		0.80(0.37-1.74)	1.02(0.54-1.94)	
Q4		1.03(0.500-2.11)	0.63(0.32-1.22)	

The dependent variable in all models is a binary variable :High-risk group vs Non-high-risk group ,with the non-high-risk group as the reference group.

a: Abbreviations: TyG, triglyceride-glucose, Q1,[5.47-8.12], Q2, (8.12-8.54], Q3, (8.54-9.01], Q4, (9.01-13.17]

Table S3 Subgroup analysis stratified by sex, age, and ethnic group examining the association between altitude, TyG index, and high risk of ASCVD.

Var	Events(n,%)	Model 4 ; OR(95%CI)			
		Q4 vs Q1	*P*-interaction	≥ 3000 vs < 2000	*P*-interaction
**Sex**					
Male	390(12.2)	**1.53(1.07-2.18)**	<0.001	**2.89(2.05-4.09)**	<0.001
Female	169(4.6)	**4.01(2.26-7.09)**		**1.81(1.05-3.12)**	
**Age**					
< 60	149(2.8)	**3.57(1.95-6.54)**	0.050	1.34(0.76-2.36)	0.017
≥ 60	410(23.9)	**1.94(1.32-2.85)**		**1.80(1.25-2.60)**	
**Ethnic**					
Han	286(8.2)	**1.21(1.38-2.83)**	0.674	**2.30(1.24-4.26)**	0.031
Minority	273(8.0)	**2.33(1.52-3.57)**		**2.68(1.83-3.94)**	
**Income**					
≤ 20000 RMB	363(9.0)	**2.61(1.81-3.76)**	0.374	**2.44(1.72-3.47)**	0.089
> 20000 RMB	196(6.9)	**1.43(1.04-2.00)**		1.59(0.96-2.63)	
**Hyptertension**					
Yes	487(16.8)	**1.60(1.14-2.25)**	0.193	**2.40(1.74-3.31)**	0.002
No	72(1.8)	**2.72(1.30-5.70)**		**2.34(1.03-5.70)**	
**DM**					
Yes	178(30.6)	1.48(0.58-3.81)	0.781	**2.91(1.53-5.52)**	0.031
No	381(6.0)	**1.43(1.01-2.01)**		**1.99(1.44-2.74)**	

The bold type represents the statistically signifcant differences ($p < 0.05$)

The dependent variable in all models is a binary variable :High-risk group vs Non-high-risk group ,with the non-high-risk group as the reference group.

Abbreviations: DM, Diabetes mellitus;

Table S4 Interaction analysis of the association between Altitude-TyG Index and high risk of ASCVD stratified by sex, age, and ethnic group on multiplicative scales.

	Sex		Age		Ethnic		Income		Hyptertension		DM	
	Male	Feamle	< 60	≥ 60	Han	Minority	≤ 20000 RMB	> 20000 RMB	Yes	No	Yes	No
**Altitude-TyG Index *P*-interaction**	0.077	0.877	0.961	0.372	0.820	0.058	0.361	0.755	0.517	0.143	0.286	0.279

Table S5 Association of TyG index and altitude with high risk of ASCVD-sensitivity analysis 1

**Levels**		**Model 1[b] OR(95%CI)**	**Model 2[b] OR(95%CI)**	**Model 3[b] OR(95%CI)**	**Model 4[b] OR(95%CI)**
**TyG[a]**	**Events**				
Q1	83	Reference	Reference	Reference	Reference
Q2	88	1.08(0.79-1.48)	1.08(0.79-1.48)	1.04(0.75-1.43)	1.09(0.79-1.50)
Q3	125	**1.48(1.10-2.00)**	**1.44(1.06-1.94)**	1.28(0.93-1.71)	1.36(0.99-1.86)
Q4	168	**1.93(1.42-2.62)**	**1.85(1.36-2.53)**	**1.42(1.01-1.99)**	**1.54(1.09-2.17)**
P for trend		<0.001	<0.001	0.023	0.007
Per IQR (0.89) increment		**1.15(1.23-1.64)**	**1.38(1.20-1.60)**	**1.23(1.05-1.44)**	**1.29(1.11-1.51)**
**Altitude**					
<2000	205	Reference	Reference	Reference	Reference
2000-2999	119	1.32(0.99-1.76)	1.29(0.96-1.72)	1.18(0.88-1.59)	1.18(0.88-1.59)
≥3000	140	**2.65(1.86-3.77)**	**2.36(1.63-3.42)**	**2.59(1.78-3.77)**	**2.71(1.86-3.95)**
P for trend		<0.001	<0.001	<0.001	<0.001
Per1-SD (631.4m) increment		**1.31(1.12-1.54)**	**1.31(1.11-1.54)**	**1.28(1.08-1.52)**	**1.30(1.09-1.54)**

The bold type represents the statistically signifcant differences ($p < 0.05$)

The dependent variable in all models is a binary variable :High-risk group vs Non-high-risk group ,with the non-high-risk group as the reference group.

a:Abbreviations: TyG, triglyceride-glucose, OR,odds ratio, Cl confdence interval, ASCVD, atherosclerotic cardiovascular diseases; Q1,[5.47-8.12], Q2, (8.12-8.54], Q3, (8.54-9.01], Q4, (9.01-13.17].

b:Model 1 was adjusted for Education, Ethnicity, Income. Model 2 was further adjusted for alcohol drinker, insufficient physical activity, insufficient vegetables and fruits. Model 3 was further adjusted for Overweight or obese, LDL-C, Hyperuricaemia, Chronic kidney disease, lipid-lowering medication, glucose-lowering medication. And Model 4 was further mutually adjusted by TyG (effect estimation of altitude;) or altitude (effect estimation of TyG)

Table S6 Association of TyG index and altitude with high risk of ASCVD-sensitivity analysis 2

Levels		Model 1[b] OR(95%CI)	Model 2[b] OR(95%CI)	Model 3[b] OR(95%CI)	Model 4[b] OR(95%CI)
**TyG[a]**	**Events**				
Q1	90	Reference	Reference	Reference	Reference
Q2	99	1.08(0.79-1.48)	1.08(0.79-1.48)	1.03(0.75-1.43)	1.09(0.79-1.50)
Q3	138	**1.48(1.10-2.00)**	**1.44(1.06-1.94)**	1.28(0.93-1.71)	1.36(0.99-1.86)
Q4	214	**1.93(1.42-2.62)**	**1.85(1.36-2.53)**	**1.42(1.01-1.99)**	**1.54(1.09-2.17)**
P for trend		<0.001	<0.001	0.023	0.007
Per IQR (0.89) increment		**1.15(1.23-1.64)**	**1.38(1.20-1.59)**	**1.23(1.05-1.44)**	**1.29(1.11-1.51)**
**Altitude**					
<2000	235	Reference	Reference	Reference	Reference
2000-2999	141	**1.93(1.39-2.66)**	**1.83(1.34-2.50)**	**1.78(1.28-2.47)**	**1.79(1.28-2.48)**
≥3000	165	**3.44(2.40-4.93)**	**3.23(2.26-4.61)**	**3.24(2.22-4.73)**	**3.49(2.39-5.11)**
P for trend		<0.001	<0.001	<0.001	<0.001
Per1-SD (631.4m) increment		**1.68(1.41-2.00)**	**1.75(1.46-2.10)**	**1.64(1.37-1.96)**	**1.67(1.39-1.99)**

The bold type represents the statistically signifcant differences ($p < 0.05$)

The dependent variable in all models is a binary variable :High-risk group vs Non-high-risk group ,with the non-high-risk group as the reference group.

a:Abbreviations: TyG, triglyceride-glucose, OR,odds ratio, Cl confdence interval, ASCVD, atherosclerotic cardiovascular diseases; Q1,[5.47-8.12], Q2, (8.12-8.54], Q3, (8.54-9.01], Q4, (9.01-13.17].

b:Model 1 was adjusted for Education, Ethnicity, Income. Model 2 was further adjusted for alcohol drinker, insufficient physical activity, insufficient vegetables and fruits. Model 3 was further adjusted for Overweight or obese, LDL-C, Hyperuricaemia, Chronic kidney disease, lipid-lowering medication, glucose-lowering medication. And Model 4 was further mutually adjusted by TyG (effect estimation of altitude;) or altitude (effect estimation of TyG)

Table S7 Association of TyG index and altitude with high risk of ASCVD-sensitivity analysis 3

**Levels**		**E-values (95%CI)**
**TyG**[a]	**Events**	
Q1	91	Reference
Q2	99	-
Q3	144	2.21(1.42-3.00)
Q4	225	3.76(2.72-7.80)
Per IQR (0.89) increment		2.06(1.77-2.35)
**Altitude**		
<2000	250	Reference
2000-2999	142	2.38(1.76-3.00)
≥3000	167	4.62(3.35-5.89)
Per1-SD (631.4 m) increment		2.28(1.90-2.66)

The dependent variable in all models is a binary variable :High-risk group vs Non-high-risk group ,with the non-high-risk group as the reference group.

a:Abbreviations: TyG, triglyceride-glucose, Q1,[5.47-8.12], Q2, (8.12-8.54], Q3, (8.54-9.01], Q4, (9.01-13.17].
